# Effects of zingerone on rat induced testicular toxicity by sodium arsenite via oxidative stress, endoplasmic reticulum stress, inflammation, apoptosis, and autophagy pathways

**DOI:** 10.22038/IJBMS.2024.73342.15934

**Published:** 2024

**Authors:** Sibel Çiğdem Tuncer, Cihan Gur, Sefa Kucukler, Serkan Ali Akarsu, Fatih Mehmet Kandemir

**Affiliations:** 1 Department of Medical Biochemistry, Faculty of Medicine, Aksaray University, Aksaray, Turkey; 2 Department of Biochemistry, Faculty of Veterinary Medicine, Atatürk University, Erzurum, Turkey; 3 Department of Reproduction and Artificial Insemination, Faculty of Veterinary Medicine, Ataturk University, Erzurum, Turkey

**Keywords:** Apoptosis, Inflammation, Oxidative stress, Sodium arsenite, Testicular toxicity

## Abstract

**Objective(s):**

This study aimed to investigate the effects of zingerone (ZNG) treatment on testicular toxicity in rats induced by sodium arsenite (SA).

**Materials and Methods:**

In the study, five groups were formed (n=7) and the experimental groups were designated as follows; Vehicle group, ZNG group, SA group, SA+ZNG 25 group, and SA+ZNG 50 group. While SA was administered orally to rats at 10 mg/kg/bw, ZNG was given to rats orally at 25 and 50 mg/kg/bw doses for 14 days.

**Results:**

As a result of the presented study, an increase was observed in the MDA contents of the testicular tissue of the rats administered SA, while significant decreases were observed in GSH levels, SOD, CAT, and GPx activities. The mRNA transcript levels of the pro-inflammatory genes NF-κB, TNF-α, IL-1β, and IL-6 were triggered after SA administration. Additionally, SA administration caused inflammation by increasing RAGE, NLRP3, and JAK-2/STAT3 gene expression. Moreover, endoplasmic reticulum (ER) stress occurred in the testicular tissues of SA-treated rats and thus ATF-6, PERK, IRE1, and GRP78 genes were up-regulated. SA caused apoptosis by up-regulating Bax and Caspase-3 expressions and inhibiting Bcl-2 expression in testicles. SA caused histological irregularities in the testicles, resulting in decreased sperm quality.

**Conclusion:**

ZNG treatment reduced SA-induced oxidative stress, ER stress, inflammation, apoptosis, and histological irregularities in the testicles while increasing sperm quality. As a result, it was observed that ZNG could alleviate the toxicity caused by SA in the testicles.

## Introduction

Heavy metals produced by natural and anthropogenic activities are common environmental pollutants (1). Sodium arsenite (SA) is one of the most dangerous environmental pollutants for human and animal health. (2). SA causes brain damage (3), cardiotoxicity (4), neurotoxicity (5), and nephrotoxic and hepatotoxic effects in rats (6). It has been reported that the arsenic compound causes dysfunction in the male reproductive system in rats and mice (7). SA suppresses spermatogenesis and testosterone release, inhibits testicular enzyme function, decreases testicular weight (8), and decreases sperm quality (9).

Heavy metals initiate a free radical-mediated chain reaction that results in lipid peroxidation, protein oxidation, and DNA and RNA oxidation. It was accepted that the administration of natural and synthetic antioxidants should be used against heavy metal poisoning (10) such as by antioxidant compounds (11). Zingerone (ZNG), one of the main components of ginger (*Zingiber officinale)* has antioxidant activity (12) and powerful pharmacological properties (13). Moreover, ZNG has anti-apoptotic (14), anti-lipidemic, antihyperglycemic (15), antimicrobial (16), anti-inflammatory (17), and anticancer activities (18). In addition, ZNG shows therapeutic properties in nephrotoxicity (19), liver, lung, testis (20), and ovarian and uterine toxicity (12).

To the best of our knowledge, there are no studies examining the possible effects of ZNG against SA-induced testicular damage. In this study, the effects of ZNG on oxidative damage, inflammation, apoptosis, autophagy in the testis caused by SA, histopathological changes in testicular tissue, and semen quality were examined in detail.

## Materials and Methods

***Animals and experimental groups***

In this study, male Sprague-Dawley rats weighing 250-300 g and aged 10-12 weeks were used. Animals were housed in the Atatürk University Medical Experimental Research Center under standard laboratory conditions (24±1 ^°^C and 45%±5% humidity, 12:12 hour dark/light cycle). Ethical committee approval was received from the Atatürk University Animal Experiments Local Ethics Committee (Protocol No:2022/237).

***Experimental groups***

Vehicle group; Oral saline was given to the rats for 14 days (n=7).

ZNG group; The rats in the ZNG group were given zingerone orally at a dose of 50 mg/kg for 14 days (n=7).

SA group; Sodium arsenite at a dose of 10 mg/kg in physiological saline was given orally for 14 days to animals in the SA group (n=7).

SA+ZNG 25 group: Firstly ZNG administered at a dose of 25 mg/kg 30 min after 10 mg/kg SA orally for 14 days (n=7).

SA+ZNG 50 group: Animals in the SA+ZNG 50 group were administered ZNG at a dose of 50 mg/kg 30 min after oral administration of 10 mg/kg SA for 14 days (n=7).

Twenty-four hours after the last SA and ZNG administration, the animals were sacrificed under mild sevoflurane anesthesia, and testicular tissues were removed and separated from the epididymis. The removed testicular tissues were divorced from the connective tissues and weighed with the help of a precision balance (Radwag R2, Germany). While the right testicle was stored at -20 ^°^C to be used in biochemical analyses, the left testicle was stored in formaldehyde solution for histopathological examination until the analysis was performed.

***Oxidative stress parameters analysis***

Testicular tissue malondialdehyde (MDA) contents were determined by the method applied by Placer *et al*. with the reaction of thiobarbituric acid (21). Glutathione (GSH) level were determined in accordance with the method used by Sedlak and Lindsay in their study (22). Superoxide dismutase (SOD) activity was performed using the method used by Sun *et al.* (23). Glutathione peroxidase (GPx) activity was measured by the method used by Matkovics *et al.* (24). Catalase (CAT) activity was measured by the method described by Aebi (25). The total protein content of the tissues was determined by the method developed by Lowry *et al*. (26).

***RT-PCR analysis***

Total RNA was isolated from testicular tissues with the help of QIAzol Lysis Reagent (79306; Qiagen). Then, cDNAs were synthesized from RNAs of all groups with the iScript cDNA Synthesis Kit (Bio-Rad). In the final stage, cDNAs β-Actin, NF-kB, TNF-α, IL-1β, IL-6, JAK-2, STAT-3 NLRP3, RAGE, Caspase-3, Apaf-1, Bax, Bcl -2, PERK, ATF-6, IRE1, GRP-78, AKT2, and FOXO1 genes were reacted with iTaq Universal SYBR Green Supermix (BIO-RAD) in a Rotor-Gene Q (Qiagen) device in the presence of reverse and forward primers. Reaction conditions were established according to the manufacturer’s instructions. At the end of the process, CT values were normalized to β-Actin. For this, the 2^-deltadeltaCT^ method was used (27). Sequences of primers are presented in Table 1.

***Histopathological examination***

Testicular tissues of the rats used in the study were fixed in 10% formaldehyde for 48 hr. Then, blocks were prepared after dehydration, clarification with xylol, and infiltration with paraffin by passing through a graded alcohol series. Sections of 5 µm thickness were obtained from the blocks with a microton and stained with Hematoxylin-Eosin (H&E). The stained sections were examined with the aid of a binocular Olympus Cx43 light microscope (Olympus Inc., Tokyo, Japan) and photographed with a EP50 camera (Olympus Inc., Tokyo, Japan). The diameter of 10 seminiferous tubules was evaluated in each animal, and the average diameter was measured in micrometers. Additionally, spermatogonia and primary spermatocytes were counted in these tubules, and the average value of each of these parameters was calculated for each group and compared with other groups (28). 

***Semen analyzes***

Sperm motility analyses were performed with a light microscope (Zeiss, Primostar, Germany) equipped on a heating plate. Ten microliters of the semen sample was dropped on the slide, and the percentage of sperm motility was determined by visual observation from 3 different microscope fields. For semen density determination, 10 µl of semen sample was mixed in an Eppendorf tube with 990 µl of eosin solution. Eppendorf tubes were vortexed for 15 seconds. Ten microliters of the mixture was taken and transferred to the Thoma chamber. Sperm cells were counted with a light microscope (Zeiss, Primostar, Germany) at 40X magnification. For analysis of abnormal sperm and dead sperm ratio, slides were stained with eosin-nigrosin solution and dried. The dried samples were then examined under a light microscope (Zeiss, Primostar, Germany) at 40X magnification. Two hundred randomly selected sperm cells were examined on each slide. Primary, secondary, and tertiary abnormalities seen in spermatozoa were examined for abnormal sperm ratio. The results were calculated as percentages. Two hundred spermatozoa were examined from the same slide and the dead spermatozoon rate was calculated. Sperms with stained heads were considered dead spermatozoons. The results were calculated as percentages (29).

***Statistical analyzes***

The data obtained from the study were statistically analyzed using the IBM SPSS program (Version 26.0), One-way analysis of variance (ANOVA) test, and the Tukey comparison test.

## Results

***Oxidative stress parameter results***

Testicular tissue oxidative stress parameter results are presented in Table 2. When the table was examined, GSH levels and GPx, CAT, and SOD activities decreased in the SA group compared to other experimental groups, while MDA levels increased (*P*<0.001). No statistically significant difference was observed between the ZNG group and the vehicle group. When the SA+ZNG 25, and SA+ZNG 50 groups were compared with the SA group, an increase was observed in GSH levels, GPx, CAT, and SOD activities, while a decrease was observed in MDA contents although not as much as high-dose (*P*<0.001).

***RT-PCR analysis results***

*Effect of SA and ZNG administrations on inflammatory genes in testicular tissue*

**Figure 1:**
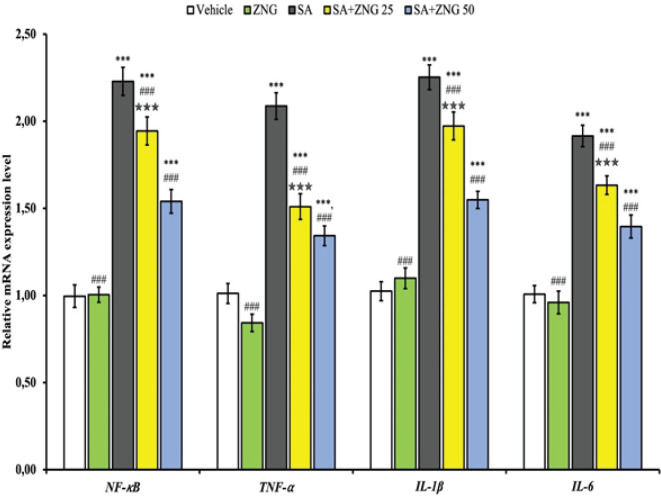
NF-κB, TNF-α, IL-1β, and IL-6 mRNA levels in testicular tissues of rats treated with sodium arsenite (SA) and zingerone (ZNG)

The mRNA transcript levels of NF-κB, TNF-α, IL1B, and IL-6, which were testicular tissue inflammatory genes of SA and ZNG administrations, are shown in Figure 1. It was observed that SA administration triggered inflammation by causing an increase in NF-κB, TNF-α, IL1β, and IL-6 mRNA transcript levels in testicular tissue (*P*<0.001). It was observed that ZNG treatment decreased the expression of NF-κB, TNF-α, IL1β, and IL-6 genes in testicular tissue in a dose-dependent manner (*P*<0.001).

***Endoplasmic reticulum stress markers in testis tissue***

**Figure 2:**
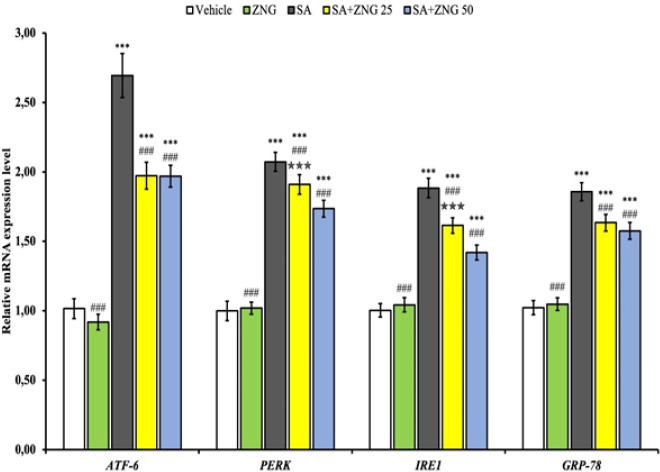
ATF-6, PERK, IRE1, GRP-78, and IL-6 protein levels in testicular tissues of rats treated with sodium arsenite (SA) and zingerone (ZNG)

The mRNA transcript levels of PERK, IRE1, ATF-6, and GRP78 genes, which are ER stress biomarkers, are given in Figure 2. It has been determined that SA causes ER stress by increasing ATF-6, PERK, IRE1, and GRP-78 in testicular tissue (*P*<0.001). On the other hand, it was observed that ZNG decreased PERK, IRE1, and GRP78 levels in a dose-dependent manner compared to the SA group (*P*<0.01), while there was no difference in ATF-6 levels between doses. There was no difference in PERK, IRE1, ATF6, and GRP-78 levels between the ZNG administration group and the vehicle group.

***Inflammation markers in testis***

**Figure 3:**
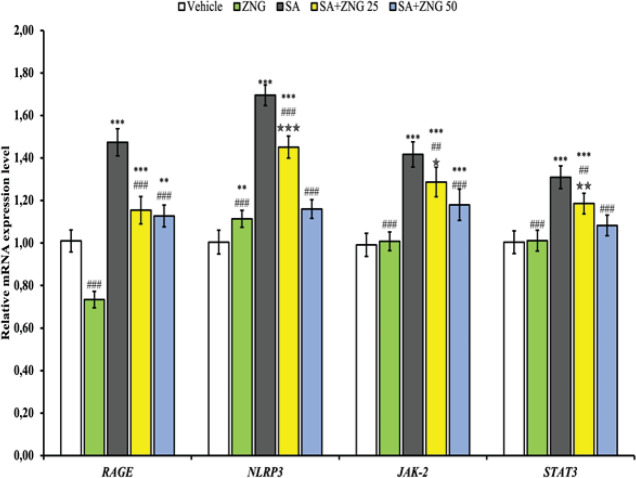
RAGE, NLRP3, JAK-2, and STAT3 mRNA transcript levels in testicular tissues of rats treated with sodium arsenite (SA) and zingerone (ZNG)

As shown in Figure 3, the levels of RAGE, NLRP3, and JAK-2/STAT 3 pathways in testicular tissue were significantly increased in the SA group (*P*<0.001). NLRP3, JAK-2/STAT3 mRNA levels decreased in a dose-dependent manner (*P*<0.001). While the RAGE levels were the lowest in the ZNG group, there were no differences between the SA+ZNG groups.

***Apoptosis markers in testicular tissue***

**Figure 4:**
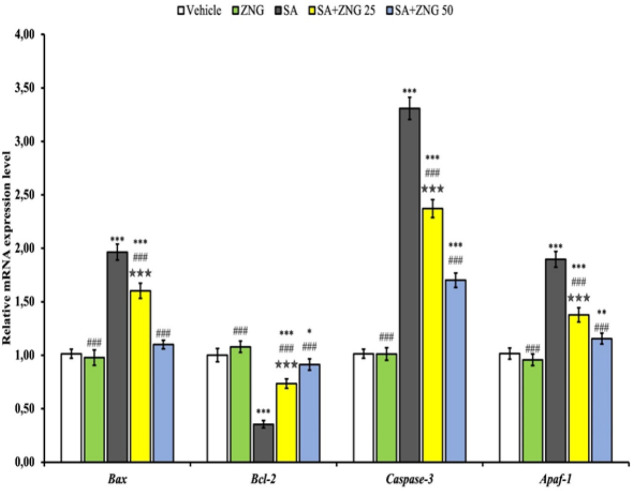
Bax, Bcl-2, Caspase-3, and Apaf-1 mRNA transcript levels in testicular tissues of rats treated with sodium arsenite (SA) and zingerone (ZNG)

The mRNA transcript levels of Bax, Bcl-2, Caspase-3, and Apaf1 in the testicular tissues of rats are presented in Figure 4. RT-PCR analysis showed that SA-induced Bax and Caspase-3 expressions suppressed Bcl-2 expression and induced apoptosis (*P*<0.001). On the other hand, treatment with ZNG provided tissue protection by disrupting this apoptotic pathway in the testis in a dose-dependent manner. There was no statistical difference between the vehicle group and the ZNG group in terms of these values.

***Akt-2 and FOXO1 pathways***

**Figure 5:**
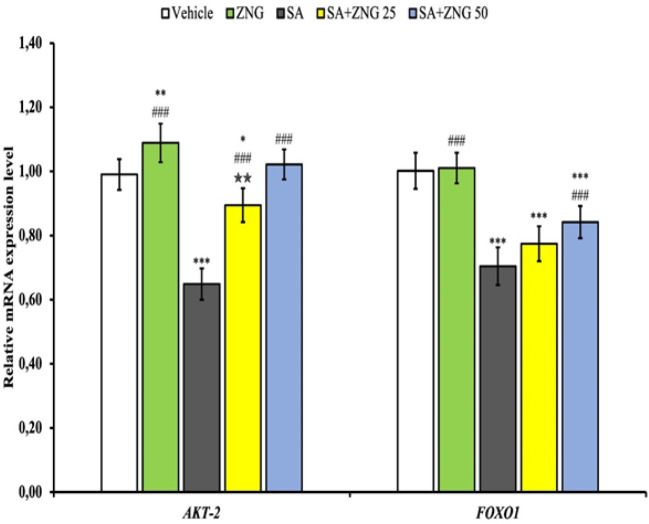
AKT-2 and FOXO1 mRNA transcript levels in testicular tissues of rats treated with sodium arsenite (SA) and zingerone (ZNG)

When AKT-2 and FOXO1 gene expressions were examined, a decrease was observed in the SA group (*P*<0.001). FOXO1 gene expressions were similar between the ZNG group and the vehicle group. Akt-2 and FOXO1 gene expression levels increased in SA+ZNG25 and SA+ZNG50 groups compared to SA treated group (*P*<0.001) (Figure 5).

***Histopathological evaluations results***

**Figure 6:**
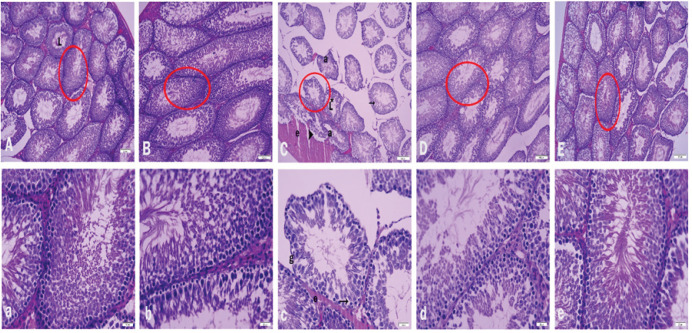
Photomicrographs of histological changes in testis tissue for experimental groups

Representative photomicrographs of the ZNG effect on SA-administered rat testis are given in Figure 6. While it was observed that the testicular tissue histologies of animals belonging to the carrier and ZNG groups showed similar morphological structures, it was determined that the seminiferous tubules of both groups were regular and their lumens were filled with sperm cells. It was observed that germ cells in the vehicle and ZNG groups were able to differentiate and had a normal structure. It was observed that tubules in rats exposed to SA showed distorted, amorphous, and atrophic images. In addition, edema and irregularities in the interstitial areas were remarkable. Vacuolization in the tubules, desquamation in the germ cells, and loss of sperm cells in the lumens were observed. There were ruptures in the germinal epithelium and basement membranes of the tubules. It was observed that ZNG treatment could minimize pathological changes depending on the dose, the tubules were healthier and the number of sperm in them increased. In addition, edema was reduced in the interstitial areas, and improvements in vacuolization were observed. Moreover, the germ cell lines were more regular, and their sizes were more normal. Rat testis morphometric findings are given in Table 3. When testicular seminiferous tubule diameters, spermatogonia, and primary spermatocytes were evaluated in the SA group, they were observed to decrease compared to the vehicle group (*P*<0.05). When the SA+ZNG 25 and SA+ZNG 50 groups were evaluated, an increase in tubule diameters, spermatogonia, and primary spermatocyte cell numbers was observed compared to the SA group (*P*<0.05) (Figure 6).

***Sperm analysis results***

The semen analysis results of the rats used in the study are presented in Table 3. The lowest total motility values were seen in the SA group and the highest in the ZNG group. It was determined that ZNG administration to the SA experimental groups had an improving effect on total motility (*P*<0.05). While the highest spermatozoon density was observed in the ZNG group, a statistical difference was found between the other groups (*P*<0.05). The SA group had the highest rate of dead spermatozoa (*P*<0.05). It was observed that ZNG administration decreased the rate of dead spermatozoa. There were no differences between the groups in terms of sperm abnormality and testicular weight. However, it was observed that right cauda epididymis weight decreased in the SA-administered groups (*P*<0.05).

**Table 1:** Primer sequences for genes

**Gene**	**Sequences (5’-3’)**	**Length (bp)**	**Accession No**
**NF-** **B**	F: AGTCCCGCCCCTTCTAAAAC R: CAATGGCCTCTGTGTAGCCC	106	NM_001276711.1
**IL-1**	F: ATGGCAACTGTCCCTGAACT R: AGTGACACTGCCTTCCTGAA	197	NM_031512.2
**IL-6**	F: AGCGATGATGCACTGTCAGA R: GGAACTCCAGAAGACCAGAGC	127	NM_012589.2
**TNF-**	F: CTCGAGTGACAAGCCCGTAG R: ATCTGCTGGTACCACCAGTT	139	NM_012675.3
**RAGE**	F: CTGAGGTAGGGCATGAGGATG R: TTCATCACCGGTTTCTGTGACC	113	NM_053336.2
**NLRP3**	F: TCCTGCAGAGCCTACAGTTG R: GGCTTGCAGCACTGAAGAAC	185	NM_001191642.1
**JAK2**	F: TAGGTACGGAGTATCTCGTG R: TGGAGTTATAGACAGCCAGG	215	NM_031514.1
**STAT3**	F: TACCTGGAGCAGCTTCATCA R: GATCTCGCCCAAGAGGTTAT	153	NM_012747.2
**Bcl-2**	F: GACTTTGCAGAGATGTCCAG R: TCAGGTACTCAGTCATCCAC	214	NM_016993.2
**Apaf-1**	F: ACCTGAGGTGTCAGGACC R: CCGTCGAGCATGAGCCAA	192	NM_023979.2
**Caspase-3**	F: ACTGGAATGTCAGCTCGCAA R: GCAGTAGTCGCCTCTGAAGA	270	NM_012922.2
**Bax**	F: TTTCATCCAGGATCGAGCAG R: AATCATCCTCTGCAGCTCCA	154	NM_017059.2
**IRE1**	F: GCAGTTCCAGTACATTGCCATTG R: CAGGTCTCTGTGAACAATGTTGA	163	NM_001191926.1
**PERK**	F: GATGCCGAGAATCATGGGAA R: AGATTCGAGAAGGGACTCCA	198	NM_031599.2
**ATF-6**	F: TCAACTCAGCACGTTCCTGA R: GACCAGTGACAGGCTTCTCT	130	NM_001107196.1
**GRP78**	F: CATGCAGTTGTGACTGTACCAG R: CTCTTATCCAGGCCATATGCAA	143	NM_013083.2
**Akt2**	F: GAGTACTTGCACTCGACGGA R: CCATGAGGATGAGCTCGAAG	304	NM_017093.1
**FOXO1**	F: CAGCCAGGCACCTCATAACA R: TCAAGCGGTTCATGGCAGAT	143	NM_001191846.3
**-Actin**	F: CAGCCTTCCTTCTTGGGTATG R: AGCTCAGTAACAGTCCGCCT	360	NM_031144.3

**Table 2:** Status of malondialdehyde (MDA), Glutathione (GSH), Superoxide dismutase (SOD), Catalase (CAT), and Glutathione peroxidase (GPx) markers in the testicular tissues of rats after sodium arsenite and zingerone administrations

**Parameters**	**Vehicle**	**ZNG**	**SA**	**SA + ZNG 25**	**SA + ZNG 50**
**MDA (nmol/g tissue)**	28.70±2.81	28.44±2.47^###^	52.86±3.12^***^	43.57±2.24^***/###/^^✯✯✯^	35.91±2.80^***/###^
**GSH (nmol/g tissue)**	6.33±0.31	6.37±0.38^###^	4.35±0.28^***^	5.04±0.22^***/##/^^✯✯^	5.60±0.22^***/###^
**SOD (U/g protein)**	17.05±1.26	16.91±1.11^###^	8.78±0.68^***^	10.25±0.71^***/^^✯✯^	12.37±1.03^***/###^
**CAT (catal/g protein)**	8.33±0.86	8.25±0.94^###^	3.45±0.46^***^	4.53±0.49^***/#/^^✯✯✯^	6.65±0.53^**/###^
**GPx (U/g protein)**	15.56±0.79	15.40±0.73^###^	6.02±0.57^***^	8.35±0.68^***/###/^^✯✯✯^	10.19±0.69^***/###^

Statistical significance; Control vs others: *P<0.05, **P<0.01, ***P<0.001, SA vs others: ^#^*P<*0.05, ^##^*P<*0.01, ^###^*P<*0.001,

SA+ZNG 25 vs SA+ZNG 50: ^✯^*P<*0.05, ^✯✯^*P<*0.01, ^✯✯✯^*P<*0.001

SA: Sodium arsenit, ZNG: Zingerone

Statistical significance (letters indicate difference between groups; *P<*0.05) analyzed using One Way ANOVA. (***P<0.001, **P<0.01, *P<0.05: Vehicle vs Others; ^###^*P<*0.001, ^##^*P<*0.01, ^#^P<0.05: SA vs Others, ^✯^*P<*0.05, ^✯✯^*P<*0.01, ^✯✯✯^*P<*0.001: SA+ZNG 25+ SA+ZNG 50)

Statistical significance (letters indicate difference between groups; *P<*0.05) analyzed using One Way ANOVA. (***P<0.001, **P<0.01, *P<0.05: Vehicle vs Others; ^###^*P<*0.001, ^##^*P<*0.01, ^#^*P<*0.05: SA vs Others, ^✯^*P<*0.05, ^✯✯^*P<*0.01, ^✯✯✯^*P<*0.001: SA+ZNG 25+ SA+ZNG 50)

Statistical significance (letters indicate difference between groups; *P<*0.05) analyzed using One Way ANOVA. (***P<0.001, **P<0.01, *P<0.05: Vehicle vs Others; ^###^*P<*0.001, ^##^*P<*0.01, ^#^*P<*0.05: SA vs Others, ^✯^*P<*0.05, ^✯✯^*P<*0.01, ^✯✯✯^*P<*0.001: SA+ZNG 25+SA+ZNG 50)

Statistical significance (letters indicate the difference between groups; *P<*0.05) analyzed using One Way ANOVA. (***P<0.001, **P<0.01, *P<0.05: Vehicle vs Others; ^###^*P<*0.001, ^##^*P<*0.01, ^#^*P<*0.05: SA vs Others, ^✯^*P<*0.05, ^✯✯^*P<*0.01, ^✯✯✯^*P<*0.001: SA+ZNG 25+ SA+ZNG 50)

Statistical significance (letters indicate the difference between groups; P<0.05) analyzed using One Way ANOVA. (***P<0.001, **P<0.01, *P<0.05: Vehicle vs Others; ^###^*P<*0.001, ^#^#*P<*0.01, ^#^*P<*0.05: SA vs Others, ^✯^*P<*0.05, ^✯✯^*P<*0.01, ^✯✯✯^*P<*0.001: SA+ZNG 25+SA+ZNG 50)

(H&E staining, original magnification; top row picture 100 µm; bottom row picture 20 µm). Vehicle group (A, a), L: Sperm in the tubule lümen, ZNG group (B, b), SA group (C, c); arrowhead: desquamation, a: atrophy, L*:sperm cell loss, e: edema, arrow: vacuolization, g: irregularity of the germinal epithelium, SA+ZNG 25 group (D,d); SA+ZNG 50

SA: arsenite, ZNG: zingerone

**Table 3:** Reproductive parameters results of experimental groups

	**Vehicle **	** ZNG**	**SA**	**SA+ ZNG 25 mg**	**SA+ZNG 50 mg**
**TM (%)**	81,88±3,22^a^	85,70±4,60^b^	62,35±3,13^abcd^	77,60±3,71^c^	83,78±4,03^d^
**Density (x 10** ^6^ **)**	73,42±6,05^a^	85,00±5,00^abcd^	70,71±7,15^b^	73,00±6,16^c^	72,42±4,79^d^
**Dead sperm**	16,28±1,60^ac^	14,28±1,25^bc^	22,57±2,50^abd^	21,57±3,04^ac^	17,42±1,81^d^
**T. A. (%)**	4,14±0,69	4,0±0,57	5,14±0,69	5,0±0,81	4,85±0,69
**H. A. (%)**	4,0±0,57	3,42±0,53	3,71±0,71	3,71±0,75	4,14±0,69
**R. T. W. (mg)**	1476,57±24,94	1431,28±76,25	1476,57±24,94	1428,00±53,87	1407,42±45,06
**L. T. W. (mg)**	1455,28±31,25	1405,0±72,91	1409,42±64,50	1403,71±60,63	1373,71±47,46
**R. C.E. W (mg)**	238,57±24,10^a^	235,71±12,72^bcd^	200,0±,8,16^ab^	204,28±9,75^c^	201,42±6,90^d^
**L. C.E. W. (mg)**	220,00±14,14	221,42±14,63^d^	197,14±11,12	202,85±9,51	200,00±5,77^d^
**Tubular diameter (μm)**	283,2±4,75	285,5±6,90	254,3±4,13	266,8±3,76	276,2±3,04
**Spermatogonia**	74,8±3,45	76±3,09	56,3±3,68	63,9±3,10	71,1±2,33
**Primary Spermatocytes**	76,9±2,60	79,2±3,08	56,1±2,13	64,8±3,01	73,3±2,54

The letters (a-d) on the same line indicate the statistical difference (*P<*0.05)

T.M: Total motility, P.M: Progressive motility, T.A: Tail abnormality, H.A: Head abnormality, R. T. W: Right testis weight, L.T.W: Left testis weight, R.C.E.W: Right cauda epididymis weight, L.C.E.W: Left cauda epididymis weight

## Discussion

Increasing heavy metal pollution causes various health problems (30). Researchers are testing compounds with various pharmacological properties against this heavy metal toxicity (31). Arsenic is a common public health problem in the environment. The damage mechanism of tissues in arsenite toxicity is generally the induction of oxidative stress (11). Moreover, the main purpose of arsenic toxicity is to inhibit the induced reactive oxygen radicals (ROS)(32). ZNG inhibits lipid peroxidation by neutralizing oxygen radicals, and as a result, it can prevent apoptosis by reducing oxidative stress (33). In the present study, the potential protective effects of ZNG on SA-induced testicular toxicity were investigated through markers of oxidative stress, inflammation, autophagy, and apoptosis.

Toxic compounds cause an increase in ROS in tissues and organs (34). ROS are governed by the balance between oxidative stress and antioxidant capacity in the male reproductive system (35). SA causes the formation of ROS and reactive nitrogen species (36). Lipid peroxidation (LPO) is one of the important markers of oxidative stress. Oxidative stress is associated with the measurement of MDA levels, which is the main indicator of LPO (37). MDA increases due to metal accumulation in the cell, and the lipid layer in the cell membrane is damaged, resulting in ROS (6). It is stated that there is an increase in MDA levels in rat testis tissue caused by SA (38). In the presented study, it was observed that while SA increased MDA level in testicular tissue, MDA level decreased with ZNG treatment in a dose-dependent manner (*P*<0.001). It is seen that antioxidant defense system enzyme levels decrease in rats administered SA, and the potential increases with ZNG treatment (*P*<0.001). It supports that this decrease is related to reducing agents, hydrogen donors, free radical quenchers, metal chelators, and redox capacities of ZNG (39).

GSH is a strong non-enzymatic antioxidant compound in a tripeptide structure, while SOD, CAT, and GPx are enzymatic antioxidants (40). The decrease in these oxidative stress defense systems causes inability to fight ROS (41). In this study, it was determined that SA inhibited SOD, CAT, and GPx enzymes, decreased GSH levels, and caused a decrease in sperm quality due to increased oxidative stress in testicular tissue. This may be due to the lack of antioxidant compounds (42). ZNG increased the activities of antioxidant enzymes and GSH levels and provided defense against oxidative stress.

High ROS levels in the testicles cause dysfunction in sperm quality and function (43). It is reported that oxidative stress is mediated by the activation of nuclear factor kappa β (NF-k β), which triggers the release of proinflammatory cytokines (44). Tumor necrosis factor alpha (TNF-α) is a major proinflammatory cytokine secreted by various cell types. TNF-α regulates spermatogenesis under physiological conditions (45). IL-1β regulates spermatogenesis and IL-6 is cytokines produced in response to infection and tissue damage (46). SA enters the nucleus of NF-κB and changes the transcription of proinflammatory factors such as IL-1β, TNF-α, and IL-6 (4). In the present study, it was determined that SA causes an increase in NF-κB protein levels and inflammation by up-regulating TNF-α, IL1β, and IL-6 gene expressions in testicular tissue. However, it is thought that ZNG reduces oxidative stress, decreases these gene expressions, and protects against inflammation.

The major endoplasmic reticulum-proximal controllers of the Unfolded Protein Response (UPR) are GRP78-triggered IRE1, PERK, and ATF-6 (47). In the literature review, it is stated that arsenic compound administration increases the endoplasmic reticulum stress (ERS) and increases PERK, IRE1α, and ATF6α cytokines (48). In mice, it increases the expression of UPR-related genes PERK, ATF6, and IRE1 by inducing ERS (49). The UPR induces apoptosis by activation of CCAAT/enhancer binding protein homologous protein (CHOP), Caspase-12, and c-Jun N-terminal kinase (50). In the presented study, SA increases PERK, IRE1, ATF-6, and GRP78 expressions in testicular tissues [*P*<0.001], which triggers the UPR, and the increase in apoptotic marker levels suggests that ER stress occurs severely. It was observed that ZNG treatment decreased ER stress by down-regulating PERK, IRE1, ATF-6, and GRP78 expressions (*P*<0.001). The decrease in apoptosis ratio of ZNG treatment can be explained by this mechanism.

JAK2/STAT3 is an important signaling pathway involved in cell proliferation, apoptosis, and inflammation (51). NLRP3 inflammation is most characterized in terms of testicular disorders (52). RAGE activates various cellular processes such as inflammation and apoptosis (53). In our study, the cytokine levels of JAK-2/STAT3, NLRP3, and RAGE increased in the SA group compared to other experimental groups (*P*<0.001). This proves that SA activates the inflammation pathway.

Proteins are divided into 3 groups according to their roles in apoptosis, apoptotic proteins, pro-apoptotic, and anti-apoptotic proteins (54). Bax protein, which is in the proapoptotic protein class, increases under oxidative stress conditions and increases the release of cytochrome C (55). Cytochrome C regulates Caspase activation by activating Apaf-1 (56). Bcl-2, an anti-apoptotic protein, maintains the integrity of the mitochondrial membrane (55). In previous studies, it has been stated that SA increases Bax and Caspase-3, which are markers of apoptosis, and decreases Bcl-2 levels in testicular tissue(9). In this study, Bax and Caspase-3 were up-regulated, while Bcl-2 mRNA transcript levels were down-regulated in testicular tissues of rats given SA. It is stated that SA increases the expression of Caspase-3 by increasing cytochrome c release (9).

The three members of the Akt family, also known as protein kinase B, are Akt1, Akt2, and Akt3 (57). FOXO is regulated by Akt phosphorylation (58). FOXOs are involved in cellular growth and organism longevity (59). FOXO1 proteins are predominantly localized in the sperm cytoplasm and Leydig cell cytoplasm in the late stages of spermatogenesis (60). In our study, the decrease in Akt-2 and FOXO1 gene expression in the SA group is evidence that apoptosis develops as a result of increased oxidative stress. The dose-dependent increase of this gene expression in ZNG groups proved that ZNG had a protective effect against SA.

SA damages the male genital tract, leading to decreased spermatogenesis and testicular testosterone production (61). SA causes arsenolysis by binding to the sulfhydryl and carbonyl groups of proteins and changing their phosphate moieties, which impairs testicular functions (62). Arsenic administration causes histopathological changes in testicles, causing damage and morphological changes in seminiferous tubules (63). In rats receiving SA, vacuoles are seen at the epithelial base in some seminiferous tubules (64). In our study, tubular degeneration, vacuolization, amorphous and atrophic images, edema, and irregularities in interstitial areas are observed in rats exposed to SA. However, histological changes were reduced to a minimal size with ZNG administration.

Andrological parameters such as sperm motility, concentration, dead-live sperm ratio, and morphology provide information about reproductive status in men (65). In a study conducted with SA, it is stated that semen density decreases and there is an increase in the number of abnormal spermatozoa (66). In another study, it was stated that sperm motility and density decreased significantly, and the rate of dead and abnormal spermatozoa increased in rats treated with SA (9). In our findings, it was observed that sperm total motility and sperm density decreased, and the rate of dead sperm and abnormal sperm rate increased in rats treated with SA (*P*<0.05). However, in general, it is seen that the quality of semen increases with the administration of ZNG in a dose-dependent manner (Table 3). In this respect, it is compatible with previous studies.

## Conclusion

As a result, it was observed that SA damaged the male genital system, increased oxidative stress and apoptosis, and decreased semen quality. By proving its antioxidant and anti-apoptotic properties, ZNG showed a dose-dependent amelioration of testicular damage caused by SA. Thus, it was observed that ZNG has potential protective properties in SA-induced testicular toxicity.

## Authors’ Contributions

SÇ T, C G, and S K designed the experiments; C G, S K, SA A, and FM K performed the experiments and collected data; SÇ T, SA A, and FM K discussed the results and strategy; SÇ T and SA A supervised, directed, and managed the study; SA A and FM K approved the final version to be published

## Conflicts of Interest

Authors declare that there are no conflicts of interest .
